# Highways*In assuming editorial charge of this Journal I felt that it would be unwise to publish my own articles of the nature usually classified as “original” in it, but that any such should be submitted to the decision of editors of other journals. In the present instance, there is an obvious conflict between this policy and the duty of publishing the program of the Central N. Y. Medical Association. The decision to publish, has been made after due consultation, taking into consideration also the fact that the subject is not one of scientific or clinical nature but one which might properly have been selected for editorial discussion.

**Published:** 1917-01

**Authors:** A. L. Benedict

**Affiliations:** Buffalo


### Highways.*

A. L. BENEDICT, A. M., M. D., Buffalo.

Presidential Address, October, 1916, Medical Association of Central New York.

Precedent is rather against the presentation of personal clinical experience in an address of this kind. The Association is too successfully following its logical purposes either to need admonition or advice or to desire praise. It is not so much modesty that prevents the solution of some great economic or ethical problem in your presence as the conviction that this would be too much like being called before a class to present a demonstration already worked out by all. Our professional problems need execution rather than solution. It has, therefore, seemed permissible to take quite literally the accepted dictum that our profession should exercise an influence in matters of general civic interest and to call attention to the subject of Highways. This is not so remote from our immediate interests as might seem. Physicians always have depended upon highways to a greater degree than members of most other professions and businesses except those directly concerned with transportation. The condition of highways not only means much to our comfort and convenience but to our efficiency. In the use of automobiles, our profession ranks second only to that of presidents, as of the country, railroads, banks and other corporations. It stands second only because such a presidency means a relatively high degree of wealth and, in actual, necessary use of automobiles, our profession stands first. Broadly considered, accidents and fatalities on the highways are as much in our domain of therapeutic and prophylactic interest as any disease. Such accidents account for 25 deaths per hundred thousand population, the 25 being divided approximately as follows: 15 caused by railroads, 4 by street cars, 5 by automobiles, 1 by other vehicles. Most of the 15 are not directly concerned with highways in the limited sense although some are, by reason of improperly planned intersections of railways and ordinary streets and roads. But at least 10 deaths per hundred thousand population per annum, or about 1 to

*In assuming editorial charge of this Journal I felt that it would be unwise to publish my own articles of the nature usually classified as "original" in it, but that any such should be submitted to the decision of editors of other journals. In the present instance, there is an obvious conflict between this policy and the duty of publishing the program of the Central N. Y. Medical Association. The decision to publish, has been made after due consultation, taking into consideration also the fact that the subject is not one of scientific or clinical nature but one which might properly have been selected for editorial discussion.

every 150 deaths from all causes, comes strictly to the charge of highways. Among our own profession, it appears that about 2% of all deaths are due to automobiles and that this proportion is more strictly vocational than in the case of the population generally. (500 deaths in J. A. M. A. gave 10 due to automobiles, besides 1 due to shot from pedestrian, many deaths not being specified as to cause).

The dangers of and limits to the practical use of automobiles are no longer due, in great measure to intrinsic imperfections of the automobile itself, nor to lack of skill and caution in its management, but mainly to inadequacy of highways. Any one of moderate physical and mental capability can drive an automobile. Even as a luxury and convenience, neither the initial expense nor the upkeep prevents its use by those of a fair degree of prosperity. It is an economic possibility for anyone who can put it to practical use for transporting passengers and merchandise, either in his regular business or as an auxiliary means of employment. The present obstacle to its more general use is mainly the lack of sufficiently spacious highways to prevent dangerous congestion and of highways kept in a passable state at all times. Even the dangers attending its use require, not so much individual caution, legislation and judicial and police inforcement of regulations as adequate streets and roads.

In quite a liberal sense, the condition is one of 18th century supply of highways confronted with 20th century demands. No fairly well settled part of the country or city dating from the latter part of the 18th century has materially added to its streets and roads. No rural or urban community or later settlement has materially improved upon the highway provision of the former, except by showing greater respect to points of compass.' In relative number, average width and expense of construction and upkeep, of highways, it is only very recently that any great change has been made from 18th century standards. Indeed, except for the increase of population and not in proportion to that, vehicular traffic was in a state of stagnation from the introduction of the steam railroad at about the beginning of the second third of the nineteenth century to the introduction of the automobile at just about the beginning of the 20th century. The introduction of the horse car at about the middle of the nineteenth century similarly discouraged urban vehicular traffic and the introduction of the electric trolley about 1890 apparently supplemented intra-urban transit for speed and inter-urban and suburban transit for frequency and fineness of network so as to comply fully with the growing demands for transportation of passengers and freight.

July, 1916, there are about 2½ million automobiles in commission, 1 to every 40 of population. By the end of the year, the number will be 3 million and the ratio 3%. N. Y. State has about 10% of both the automobiles and the population of the U. S. In Iowa and centain sections of other states the ratio is 1:15 and the unchecked sales indicate that this ratio or even a 1:10 ratio may be reached in the near future. By the end of the present decade, we may expect a demand on highways equivalent to what would have gradually developed if rail transportation had never been invented. The great bulk of this demand will have developed in ten years instead of 80. This statement apparently ignores the fact that rail transportation, in some form does and undoubtedly will continue to care for the vast majority of long distance freight and passengers and for a large minority, at least, of short distance passenger traffic, especially in inclement seasons. But, on the other hand, the history of our country in the first half of the nineteenth century and contemporary conditions in Europe indicate that water routes, natural and artificial, would unquestionably have been developed as a partial substitute for rail transportation and it must not be forgotten that while rail transportation successfully competes with the motor for long distance and large bulk, it also indirectly creates a demand for the latter for short distance and small bulk. Or, more broadly stated, the railroad has rendered possible a degree of movement of human beings and freight that could not have developed by vehicular and boat traffic.

The stagnation of the highways for some 80 years involves political as well as economic features. The nation has, practically, never exercised its explicit constitutional control over post roads nor its implicit powers over roads for military purposes. The states have failed to assume authority and responsibility regarding highways supplemental to the nation, as they inevitably would have done if the railroads had not usurped the functions of the people in providing routes of intercourse. Thus, street and road provision and maintenance have devolved almost entirely on local governments and have been neglected except in cities to a large degree, until very recently. Country roads are in fair condition for only a quarter of the days of a year and a village or city in its infancy is wet most of the time.

At this point, an amazing admission must be made. The number, distribution and width of rural highways, as laid out in the eighteenth century or in the nineteenth century but with no anticipation of 20th century developments, are, with comparatively minor exceptions, adequate to present needs and those of the near future. It is only the road way itself that requires extensive improvement. The original area of most of our cities also remains adequate to the demands of the same area if it were still an entire city. Even as the business center of a much extended municipal area, the streets would be fairly adequate to present demands if the original plan of the city had been extended systematically to meet growth of population. The main defect in most cities is that they have grown by increments of pre-existing independent villages or of artificial real estate syndicates and that no or few direct through routes have been laid out in addition to the former country roads connecting them with each other and with the down-town district corresponding to the original city. These trunk streets have, of course, been preempted for street railways so that, under present conditions, dangerous congestion of traffic occurs and there is a constant loss due to the diversion of traffic by round-about routes from one part of the city to another. In Buffalo, for example, there is less than one exit per mile of boundary; only two exits not encumbered by street car tracks and one of these leading no where in particular; only four streets continuous from city line to city line and two of these merely diagonally for a comparatively short distance across a corner of the city. The original village of Buffalo constitutes the south-western corner of the present city and the Lake and River do away with traffic demands along its south-western front, yet in some way, we have perversely accomplished the apparently impossible result of compelling most extra-urban and a large part of interurban traffic which would gladly avoid the congested business center, to pass through it.

The fact should be clearly comprehended and promptly acted upon that the dangers of street congestion cannot be removed by traffic ordinances but only by an adequate provision of through streets and that, in the long run, such provision is more economic than the increasing expense of traffic police, the damage to property and person inevitable under existing conditions and the expense of time and mileage in following devious routes. The problem does not require in all or even many instances the enormous expense of razing buildings and creating parallel thoroughfares. A single block of rough pavement may interrupt an available thoroughfare. Others really exist but are scarcely known to residents, being composed of a series of short streets which require only rounding off of corners at jogs, occasionally a cut through back-yard property and some paving, to make a practically straight trunk route. Slight changes in street car routes, widening between curbs, compelling a few firms to park delivery wagons on their own ground floors or in basements and similar minor improvements will often solve the problem much more perfectly and more cheaply than might be imagined.

The problem of rural roads is really of greater practical importance generally and to our own profession than that of city streets, since in the latter instance, trolley transit remains fairly adequate except as a matter of luxury and personal convenience. The problem is also limited mainly to the matter of road bed, including necessary engineering factors as of grades and bridges. Such new routes or connections of existing routes as are required do not involve the enormous expense for land involved in similar urban problems.

A great deal has been done toward rendering country roads available as practical routes of travel and transportation within the last few years but much remains to be done and it can be done successfully only by the assumption of power and responsibility by the nation and states, to insure systematic development. For example New York State has about 80,000 miles of rural highways, about 6000 being macadamized, bricked or cemented, and 1300 miles more being under construction. Without going into details, it can be shown that 2700 miles of improved highway would provide for roads encircling the state, for two through east and west highways—all that are practicable when we consider the obstacles afforded by the lake system—and for north and south highways at intervals of about 30 miles. Such a system would connect, fairly directly, almost all cities and towns of 10,000 population and over and, indeed, almost all centers of population except the smallest. Yet, with almost three times as much improved roads, we have only the following trunk highways: one east and west from Buffalo to Albany, almost constantly interrupted for repairs; one very devious east and west southern route, not completed at either end though a person with ample time can tap it by way of Avon and eventually reach the Hudson River and even New York City; a north and south route at the eastern, long edge of the state, decidedly sinuous north of Albany; a few north and south transepts, fairly straight and reaching somewhere near the northern and southern boundaries of the state. With these exceptions, it is impossible to start from any point in the state and proceed safely to any other point at a distance of 50 miles. Except from Albany and New York City, near the extreme eastern edge of the state, our logical share in the development of natural interstate highways has not been performed. About half of the N. Y. interstate highway S. W. from Buffalo is in bad condition. In the last two years military preparedness and the necessary part of highways have been much discussed. Contrast the actual with the possible provision of practical thoroughfares from this standpoint alone. At the beginning of highway improvement, the necessity of scattering object lessons over the state, was obvious. At present, even from the standpoint of impartiality, the average resident of a given town might much better have a missing link filled in at a distance of 50 to 100 miles than have an additional stub of good country road in his immediate vicinity.

With individual exceptions, experience has proved that the macadam road does not meet existing demands. It is not only short-lived but subject to frequent attacks of calculus disease and greasy degeneration. Macadam did not anticipate the rubber tired vehicle and it is an injustice to his memory to apply his name to a mixture of Indian arrow heads and crude oil. The relatively permanent brick and cement road beds are in the end, cheaper than so-called macadam, the average cost being $25,000, $15,000 and $10,000 per mile. The first two can be repaired without serious interruption of traffic. The macadam road, as contrasted not only with brick, cement and asphalt but even with sand, gravel, shale and clay imposes an indirect tax in tire damage fully equal to the license tax, from which, at present, is derived about 2 of the 10 million dollars expended for road improvement and maintenance in the State of N. Y.

One cannot make a road and use it too but it is certainly possible to make repairs in such sections and lateral halves as to avoid further interruptions of traffic. It is conceivable that a concrete bridge might be completed in the time required for the foundation of a 75 c.m. cannon and a road constructed without blocking any part of it for two seasons or delaying the construction of a natural unit of thoroughfare for five or six years. Improved roads as stated cost $10,000-$25,000 a mile: the additional expense of $500 to make necessary detours passable by dumping natural gravel or shale on the low parts, would not appreciably increase the expense. Theoretically and perhaps practically, it would be possible for the state to buy a few miles of plank and construct safe, if not ideal temporary roadways alongside construction work, although the judgment of the Commissioner of Highways is against this suggestion. It may even be questioned whether, for a few years, it would not be better to limit new construction to the comparatively few miles of missing links necessary to complete the system of trunk highways already alluded to, and to spend whatever remains of its funds in grading the worst hills, and in dumping sand, gravel and shale—not cracked corniferous limestone—on the worst stretches of clay road. It is a matter of common sense not to close adjacent, parallel routes at the same time, yet this is repeatedly done both in the country and in the city. Three years ago, every possible outlet from Buffalo was obstructed by some form of improvement, except one street, in bad condition which led to no definite destination.

If road improvement is undertaken at all, no reasonable expense should be spared to avoid danger. For example, the Niagara Falls Boulevard, is a bait to every automobilist in the country to enter a succession of death traps. The loss of life and damage to property already incurred on this 20-mile course, balance any conceivable initial expense to insure a freedom from dangerous curves or an additional width commensurate with the amount of travel. The majority of overhead or underground railway crossings, are, on account of grade, narrowness and compound curves employed in their approach, more dangerous than a crossing at grade in the open.

It should be understood that anything in the way of criticism, here presented, is by no means directed against the Highway Commission. On the contrary, this Commission has shown the utmost courtesy in furnishing data and has accomplished wonders in the short time of its existence, especially when we consider that the problems with which it has been confronted are novel and that their solution has been aided only in a very general way by available experience. The principal difficulty seems to be that lack of centralized and coordinated authority characteristic of this country and perhaps impossible of attainment in any country which is fundamentally democratic.

---

**Radium Treatment of Cancer of the Cervix.** Palmer Findley, Omaha, Am. Jour. of Surg., Nov., reports 9 cases. All but one were over 48, most in the fifties, but one was 78. One girl of 23 showed advanced cancer. Contrary to what is sometimes taught, excessive child-bearing does not seem to have been a factor. 3 were nulliparae, 2 biparae, 2 triparae, 1 quinquepara, 1 not stated. Three cases regarded as inoperable became operable. Two are alive, one without evidence of return after 10 months, the other with evidence of return after 14 months. In one case radium failed to influence even superficial parts of the growth, and in 3 it was thought that death might have been hastened. In 2 cases, pain was relieved, in 4 only partially or not all. The author is, however, favorably inclined toward radium.

---

**Wandering Tapeworm.** Alfred Dean of Grand Forks, N. Dak., Ther. Gaz., Oct., reports a case of abscess over the iliac cest, apparently faecal from the odor, from which 16 segments of tapeworm were removed in two weeks, beginning one week after the first incision. The patient then discharged 17 feet by bowel, spontaneously.

